# Amantadine Did Not Positively Impact Cognition in Chronic Traumatic Brain Injury: A Multi-Site, Randomized, Controlled Trial

**DOI:** 10.1089/neu.2018.5767

**Published:** 2018-09-24

**Authors:** Flora M. Hammond, Mark Sherer, James F. Malec, Ross D. Zafonte, Sureyya Dikmen, Jennifer Bogner, Kathleen R. Bell, Jason Barber, Nancy Temkin

**Affiliations:** ^1^ Department of Physical Medicine and Rehabilitation, Indiana University School of Medicine, Indianapolis, Indiana.; ^2^ Rehabilitation Hospital of Indiana, Indianapolis, Indiana.; ^3^ Department of Physical Medicine and Rehabilitation, Carolinas Rehabilitation, Carolinas HealthCare System, Charlotte, North Carolina.; ^4^ TIRR Memorial Hermann, Houston, Texas.; ^5^ Spaulding Rehabilitation Hospital, Boston, Massachusetts.; ^6^ Department of Rehabilitation Medicine, University of Washington, Seattle, Washington.; ^7^ Department of Physical Medicine and Rehabilitation, The Ohio State University, Columbus, Ohio.; ^8^ Department of Physical Medicine and Rehabilitation, University of Texas Southwestern Medical Center, Dallas, Texas.; ^9^ Department of Neurological Surgery, University of Washington, Seattle, Washington.; ^10^ Department of Biostatistics, University of Washington, Seattle, Washington.

**Keywords:** amantadine, attention, brain injuries, cognition, executive function, memory

## Abstract

Despite limited evidence to support the use of amantadine to enhance cognitive function after traumatic brain injury (TBI), the clinical use for this purpose is highly prevalent and is often based on inferred belief systems. The aim of this study was to assess effect of amantadine on cognition among individuals with a history of TBI and behavioral disturbance using a parallel-group, randomized, double-blind, placebo-controlled trial of amantadine 100 mg twice-daily versus placebo for 60 days. Included in the study were 119 individuals with two or more neuropsychological measures greater than 1 standard deviation below normative means from a larger study of 168 individuals with chronic TBI (>6 months post-injury) and irritability. Cognitive function was measured at treatment days 0, 28, and 60 with a battery of neuropsychological tests. Composite indices were generated: General Cognitive Index (included all measures), a Learning Memory Index (learning/memory measures), and Attention/Processing Speed Index (attention and executive function measures). Repeated-measures analysis of variance revealed statistically significant between-group differences favoring the placebo group at day 28 for General Cognitive Index (*p* = 0.002) and Learning Memory Index (*p* = 0.001), but not Attention/Processing Speed Index (*p* = 0.25), whereas no statistically significant between-group differences were found at day 60. There were no statistically significant between-group differences on adverse events. Cognitive function in individuals with chronic TBI is not improved by amantadine 100 mg twice-daily. In the first 28 days of use, amantadine may impede cognitive processing. However, the effect size was small and mean scores for both groups were generally within expectations for persons with history of complicated mild-to-severe TBI, suggesting that changes observed across assessments may not have functional significance. The use of amantadine to enhance cognitive function is not supported by these findings.

## Introduction

Long-term cognitive impairment has been reported in up to 65% of individuals with moderate-severe traumatic brain injury (TBI) with adverse effects on independence, homemaking tasks, interpersonal relationships, leisure, employment, and other aspects of life.^1^ In spite of limited evidence of efficacy, prescribing pharmacological agents to improve chronic cognitive dysfunction after TBI is common practice. The dopaminergic agent and N-methyl-D-aspartate (NMDA) antagonist, amantadine (approved by the U.S. Food and Drug Administration for influenza prevention and Parkinson's disease), is commonly used for this purpose.^2^ Amantadine has a relatively benign side-effect profile (assuming adequate renal function) compared to other agents. Although previous studies are limited by small sample size and design flaws, preliminary evidence suggests some cognitive benefit from amantadine. Notably, there is strong evidence that amantadine improves rate of recovery acutely in those with severe TBI and prolonged disorder of consciousness.^3,4^

Kraus and colleagues^5^ found a positive effect of amantadine on executive function, but not attention and memory, in an open-label, prospective study of 22 individuals with chronic TBI receiving 400 mg of amantadine daily over 12 weeks. A retrospective study by Reddy and colleagues^6^ studied amantadine use in 25 adolescents whose cognitive function failed to return to baseline after 21 days of rest after sports-related concussion. This cohort who received amantadine 100 mg twice-daily was compared to a cohort (matched on age, sex, and concussion history) who were not treated with pharmacological agents. This comparison found superior pre- to post-test improvements in concussion symptoms (total score on 22-item self-report symptom inventory), verbal memory, and reaction time for the amantadine group, but no between-group differences for visual memory and visual motor processing speed. In contrast, a small (*n* = 10), double-blind, placebo-controlled, cross-over study demonstrated no differences in the rate of cognitive improvement (orientation, attention, executive function, memory, behavior, and a composite variable) between patients treated with amantadine (100–300 daily) and placebo during acute inpatient rehabilitation.^7^ In a systematic literature review on dopaminergic agents for cognition and agitation, Leone and colleagues^8^ concluded that amantadine may be a reasonable option to improve cognition and reduce agitation after a TBI, but confirmatory evidence is needed. Despite the limited evidence to support amantadine use to enhance cognitive function after TBI, clinical use for this purpose is highly prevalent and is often based on inferred beliefs. This common practice has been demonstrated through data collected on prescribing patterns^9,10^ and a survey of brain injury physicians, which indicated that amantadine is a popular pharmacological choice for treating memory impairment, inattention, slow mental processing, and abulia.^11^

Given the common use of amantadine to enhance cognitive performance after TBI and the limited evidence of efficacy, there is a need for rigorous scientific evaluation of amantadine's effect on cognition. The Amantadine Irritability Multi-site Study (AIMS) was a large, multi-site, randomized, placebo-controlled study performed to assess the effect of amantadine on irritability (primary outcome) and aggression, anger, and cognitive function (secondary outcomes).^12^ The current study investigated the effect of amantadine on cognitive function for participants in the AIMS study. Based on past literature, we hypothesized that, compared to placebo, amantadine (100 mg every morning and noon) would significantly improve cognitive function (overall cognitive performance, processing speed, memory, attention, and executive function) from baseline assessment to days 28 and 60.

## Methods

### Setting

Study sites were: Carolinas Rehabilitation in Charlotte, North Carolina (lead site); Rehabilitation Hospital of Indiana (Indianapolis, IN); Kessler Institute of Rehabilitation (West Orange, NJ); Spaulding Rehabilitation Hospital (Boston, MA); TIRR Memorial Hermann (Houston, TX); The Ohio State University (Columbus, OH); and University of Washington (Seattle, WA).

### Study oversight

Institutional review board approval was received at each site, informed consent obtained from all participants and their observers, and the study registered on www.clinicaltrials.gov (#NCT00779324). An external Data and Safety Monitoring Board provided independent oversight. A data coordinating center (DCC) managed the concealed treatment allocation, data storage, and data monitoring and, upon study closure, transferred the data to the statistician.

### Participants

Recruitment was through referrals, physician letters, and newsletters. Individuals were eligible if 16–75 years of age, sustained a nonpenetrating TBI at least 6 months prior to enrollment, and obtained a score ≥6 on observer-rated Neuropsychiatric Inventory–Irritability. TBI was verified by record review and clinician interview as meeting at least one of the following criteria: 1) post-resuscitation Glasgow Coma Scale (GCS) <13; 2) GCS Motor <6 off paralytics; 3) loss of consciousness attributable to TBI; 4) post-traumatic amnesia lasting ≥24 h; 5) neuroimaging consistent with TBI; and/or 6) other evidence of TBI-related focal neurological findings. An observer (family member, close friend, or employer) with adequate interaction to observe irritability was required. Enrollment was further contingent upon medical and neurological stability, ability to comply with study protocol, negative pregnancy test, and creatinine clearance >60. Individuals were excluded if: 1) unable to interact and communicate; 2) threat of harm to self or other; 3) history of neurologic disorder, schizophrenia, or psychosis; 4) seizure in month preceding enrollment; 5) concomitant use of typical neuroleptic agents or monoamine oxidase inhibitors (because of potential drug interactions); and 6) amantadine ingested during the month preceding enrollment. All medications were on stable dosing for at least 1 month before enrollment with no plans to change medications during the 60-day study. Active rehabilitation therapies, behavior treatments, and counseling, if present, were started at least 1 month before enrollment, and none were started during the study. We did not attempt to record and compare groups on therapy involvement because both rehabilitation and psychological therapies may vary considerably in approach and quality among providers with no accepted method to quantify therapy impact. We relied on random assignment to control for this and other potential group differences.

A sample of 168 was enrolled based on the sample needed to replicate the study of the effect of amantadine on the primary outcome (irritability).^12^ From the 168 study participants, a subset of 119 was selected with significant cognitive impairment as indicated by two or more cognitive test scores at least 1 standard deviation (SD) below normative values. Of this subset, 59 were in the amantadine group and 60 in the placebo group.

### Procedures

Demographic, medical history, and injury data were collected and verified through interview and record review. Measures of participant mood and behavior were administered to the participant and the participant's observer. Cognitive performance tests were administered by trained study coordinators and audio recorded for quality assurance review. After confirmation of eligibility and baseline assessment, participants were randomly allocated 1:1 to take amantadine (100 mg every morning and noon) or placebo equivalent. In cases of presumed drug intolerance, the dose was reduced or terminated per pre-specified protocol. Participants and observers completed the assessment measures at baseline and treatment days 28 and 60.

### Randomization and masking

The DCC conducted computer-generated block randomization and concealed group allocation. After the site coordinators entered the participant's eligibility data into the study webpage and eligibility confirmed, the DCC assigned a study number that indicated which study drug kit to dispense. Randomization was stratified on presence of depression at time of enrollment (<13 vs. ≥13 on the Beck Depression Inventory [BDI]–II)^13^ based on possible association of depression with irritability. The compounding pharmacist and DCC had access to group assignment; all other study personnel, participants, and observers were blinded to group allocation.

### Measures

A battery of neuropsychological measures with well-established psychometric properties was used^14–19^ (see Table 1). Scores from these measures were converted to standardized scores using the appropriate norm sets.

**Table 1.** Neuropsychological Test Battery

*Measure*	*Cognitive functions measured*
Digit Span, Wechsler Memory Scale–III^14^	Attention, working memory
Trail Making Test^15^	Alternating attention, visual-motor coordination
Controlled Oral Word Association Test (COWAT)^16^	Verbal fluency, executive function
California Verbal Learning Test-II (CVLT-II)^17^	Verbal learning and recall
WAIS-III Processing Speed Index (PSI; comprised of Digit Symbol and Symbol Search)^18,19^	Attention, visual-motor coordination, and psychomotor speed

#### Other measures

Data were collected to characterize the sample (see Table 2).

**Table 2.** Comparison of Demographic and Injury Characteristics by Treatment Group

		*Treatment group*		
*Characteristic*	*Overall*	*Placebo*	*Treatment*	*Sig.*
Subjects	119	60	59	
**Age, years**				
**Mean (SD)**	38.6 (12.4)	37.4 (12.2)	39.9 (12.6)	0.258
<40	67 (56%)	34 (57%)	33 (56%)	
≥40	52 (44%)	26 (43%)	26 (44%)	
Years post-injury				
Mean (SD)	6.2 (5.5)	6.0 (5.1)	6.3 (6.0)	0.823
<5	64 (54%)	34 (57%)	30 (51%)	
≥5	55 (46%)	26 (43%)	29 (49%)	
**Race**				
White	103 (87%)	52 (87%)	51 (86%)	1.000
Black	9 (8%)	4 (7%)	5 (8%)	
Other	7 (6%)	4 (7%)	3 (5%)	
**Education years**				
Mean (SD)	13.3 (2.1)	13.3 (2.2)	13.3 (1.9)	0.983
A: Less than HS	11 (9%)	7 (12%)	4 (7%)	
B: High school/GED	42 (35%)	19 (32%)	23 (39%)	
C: Some college	49 (41%)	26 (43%)	23 (39%)	
D: 4yr+ degree	17 (14%)	8 (13%)	9 (15%)	
**Primary activity**				
Student	8 (7%)	3 (5%)	5 (8%)	0.332
Competitively employed	17 (14%)	11 (18%)	6 (10%)	
Retired	51 (43%)	25 (42%)	26 (44%)	
Unemployed	32 (27%)	18 (30%)	14 (24%)	
Other	11 (9%)	3 (5%)	8 (14%)	
**Post-traumatic amnesia**				
0: None	5 (4%)	2 (4%)	3 (5%)	0.709
1: 1–30 min	3 (3%)	2 (4%)	1 (2%)	
2: >30 min to <1 day	1 (1%)	0 (0%)	1 (2%)	
3: ≥1 day	104 (92%)	53 (93%)	51 (91%)	
Unknown	6	3	3	
**Loss of consciousness**				
0: None	9 (8%)	6 (10%)	3 (5%)	0.192
1: 1–30 min	11 (10%)	6 (10%)	5 (9%)	
2: >30 min to <1 day	14 (12%)	9 (15%)	5 (9%)	
3: ≥1 day	81 (70%)	39 (65%)	42 (76%)	
Unknown	4	0	4	
**Depression**				
BDI <13	38 (32%)	19 (32%)	19 (32%)	1.000
BDI ≥13	81 (68%)	41 (68%)	40 (68%)	
				

SD, standard deviation; HS, high school; GED, General Educational Development; BDI, Beck Depression Inventory.

### Statistical analysis

Analyses were performed using SAS^®^ statistical software (version 9.3; SAS Institute Inc., Cary, NC).^20^ A two-tailed *p* value <0.05 was considered statistically significant.

Cognitive performance composites were constructed by converting each individual score to a sample percentile (i.e., the rank divided by the number of available scores) and calculating the mean percentile for each subject across all relevant measures.^21^ A high score corresponded to a good outcome for all measures and composites. (Note: Due to a few missing values, the overall sample mean for each composite does not equal exactly 50.) The following indices were created:

- **Learning Memory Index (LMI):** Comprised of the following California Verbal Learning Test (CVLT) measures: Trials 1–5 Total Score (T-score), Short Delay Free Recall (Z score), Short Delay Cued Recall (Z score), Long Delay Free Recall (Z score), and Long Delay Cued Recall (Z score).
- **Attention/Processing Speed Index (APSI):** Comprised of Digit Span (Scaled Score), Processing Speed Index, Trail Making Test–A (T Score), Trail Making Test–B (T score), and Controlled Oral Word Association Test (COWAT; T score).
- **General Cognitive Index (CGI):** Comprised of measures in LMI and APSI.
- **Baseline Cognitive Index (BCI):** Baseline CGI.

Treatment groups were compared on baseline characteristics and BCI. For ordinal variables, the normal approximation for the Wilcoxon rank-sum test was used. For categorical variables, Fisher's exact tests were used.

Analyses were performed using the intention-to-treat principle. Pre-specified outcome analyses included: 1) comparison of the change in cognitive performance scores from baseline to day 28 and baseline to day 60 and 2) comparison of the change in cognitive indices (GCI, LMI, and APSI) from baseline to day 28 and baseline to day 60 using linear mixed-effects modeling, where each subject was modeled with a random slope and intercept, time was treated as categorical, and no other covariates were included in the models.

## Results

### Participants

**FIG. 1.**
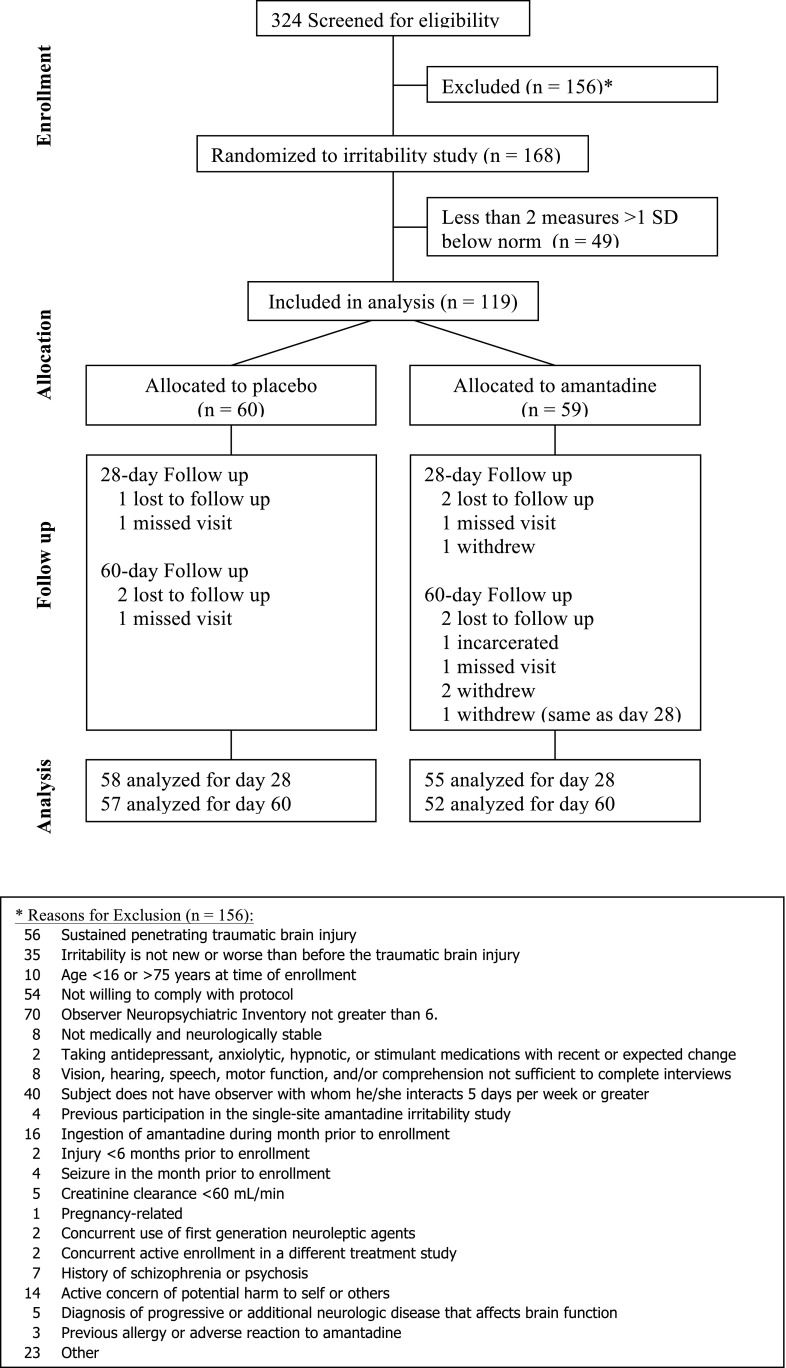
Participant flow diagram. SD, standard deviation.

From August 2009 to April 2013, 168 individuals (enrollment target for irritability outcome) were enrolled and randomized, with 119 meeting criteria for cognitive impairment (60 placebo and 59 amantadine) as outlined in Figure 1.^8^ Compliance, defined as ≥80% prescribed study drug consumed per pill count, was high (88.5% in amantadine group and 86.9% in placebo group). Eleven participants (6.6%; 4 placebo and 7 amantadine) did not complete the study. On rare occasions, study participants were unable to complete the cognitive tests (e.g., inability to present in-person). Supplementary Digital Content Table (see online supplementary material at http://www.liebertpub.com) provides an inventory of neuropsychological measure availability (sample size) for each treatment group by assessment interval.

Table 2 summarizes and compares the baseline demographic and injury characteristics of the amantadine and placebo groups. Groups were well matched with respect to baseline factors. Table 3 compares the baseline scores for the neuropsychological test variables by treatment group. Baseline scores for the placebo group were slightly lower than that for the treatment group, but only PSI was nominally significant (*p* = 0.048).

**Table 3.** Comparison of Neuropsychological Test Variables by Treatment Group at Baseline and Linear Modeling of All Outcome Measures for the Cognitively Impaired Sample (*n* = 119)

		*Raw scores*				*Linear model*^a^		
*Outcome measure*	*Day*	*Placebo*	*Treatment*	*Difference*	*Change from baseline*	*Model-adjusted change*	*Sig.*	*95% CI*
Rey (T)	0	34.5	37.7	3.16				
	28	41.7	41.1	–0.58	–3.74	–3.62	0.045	(−7.16, −0.08)
	60	43.5	46.9	3.45	0.29	–0.46	0.801	(−4.05, 3.13)
Short Delay Free (Z)	0	–1.43	–1.32	0.11				
	28	–0.94	–1.01	–0.07	–0.18	–0.21	0.231	(−0.55, 0.13)
	60	-0.77	–0.63	0.15	0.04	–0.06	0.720	(−0.41, 0.29)
Short Delay Cued (Z)	0	–1.43	–1.30	0.13				
	28	–0.91	–1.01	–0.10	–0.22	–0.25	0.154	(−0.58, 0.09)
	60	–0.71	–0.60	0.11	–0.01	–0.12	0.482	(−0.47, 0.22)
Long Delay Free (Z)	0	–1.66	–1.45	0.21				
	28	–1.00	–1.16	–0.16	–0.37	–0.40	0.020	(−0.73, −0.06)
	60	–0.88	–0.79	0.09	–0.12	–0.24	0.171	(−0.58, 0.10)
Long Delay Cued (Z)	0	–1.58	–1.44	0.13				
	28	–0.95	–1.08	–0.13	–0.27	–0.28	0.074	(−0.59, 0.03)
	60	–0.71	–0.64	0.06	–0.07	–0.20	0.216	(−0.51, 0.12)
Digit Span	0	8.47	8.75	0.28				
	28	9.02	9.07	0.06	–0.22	–0.14	0.670	(−0.76, 0.49)
	60	8.95	9.27	0.33	0.05	0.13	0.682	(−0.50, 0.77)
PSI	0	78.5	83.0	4.53				
	28	83.6	85.9	2.34	–2.19	–1.33	0.299	(−3.85, 1.19)
	60	84.5	88.8	4.36	–0.17	0.14	0.914	(−2.42, 2.71)
Trail A (T)	0	36.4	39.8	3.46				
	28	40.3	42.0	1.69	–1.78	–0.85	0.625	(−4.25, 2.56)
	60	41.9	45.0	3.14	–0.32	–0.25	0.888	(−3.71, 3.22)
Trail B (T)	0	37.6	39.5	1.93				
	28	41.4	41.0	–0.35	–2.28	–1.20	0.522	(−4.91, 2.50)
	60	40.1	42.5	2.34	0.41	0.37	0.846	(−3.38, 4.13)
COWAT	0	37.6	36.3	–1.36				
	28	38.8	38.5	–0.26	1.10	1.34	0.251	(−0.95, 3.63)
	60	39.5	39.9	0.42	1.79	2.12	0.074	(−0.21, 4.45)
Overall Composite (GCI)	0	47.3	52.7	5.37				
	28	50.6	49.2	–1.39	-6.76	–6.24	**0.002**	(−10.14, −2.34)
	60	48.3	51.9	3.58	–1.79	–2.62	0.195	(−6.59, 1.35)
Learning/ Memory Index (LMI)	0	46.7	53.4	6.69				
	28	51.5	48.3	–3.20	–9.90	–10.16	**0.001**	(−16.32, −4.00)
	60	48.4	51.9	3.49	–3.20	–5.37	0.093	(−11.63, 0.90)
Attention/ Processing Speed Index (APSI)	0	48.0	52.0	4.04				
	28	49.6	50.2	0.57	–3.46	–2.11	0.250	(−5.71, 1.49)
	60	48.2	51.9	3.67	–0.36	0.26	0.889	(−3.41, 3.93)

Bolded font indicates that the result remained statistically significant (*p* < 0.05) after adjusting for multiple comparisons (Holm-Bonferroni, m = 26).

^a^ Mixed-effects linear regression model, treating subject as random and time as categorical, with no other model covariates. Reported model statistics correspond to the time-by-treatment interaction. Positive change scores indicate a positive treatment effect.

CI, confidence interval.

### Treatment group comparisons

The unadjusted standardized scores and change from baseline are displayed in Table 3 sorted by assessment interval and treatment group. At day 28, the mean change for the placebo group demonstrated greater improvement than for the amantadine group for all the measures except Digit Span and COWAT. Because change from baseline was apparent at day 28 on the memory measures but not the nonmemory measures, we examined the degree of change on individual memory tests included in the CVLT. A high percent of those in the placebo group showed positive change from baseline to day 28 (59–65%) compared to the amantadine group (46–53%) on all CVLT measures. Note that except for baseline measures in the placebo group, all scores for both groups were within normal limits defined as within 1 SD of the normative group mean.

Results of the mixed-effects linear regression modeling of the cognitive indices are summarized in Table 3. Greater improvement in GCI (6.2 percentiles; 95% confidence interval [CI], 2.3–10.1; *p* = 0.002) and LMI (10.2; 95% CI, 4.0–16.3; *p* = 0.001) was found for the placebo group compared to the amantadine group at day 28. Improvement in APSI did not significantly differ between the groups (*p* = 0.250). At day 60, no significant between-group differences were observed for any of the indices.

### Adverse events

Amantadine was well tolerated among study participants with no significant between-group differences on withdrawals/lost or adverse events (using Fisher's exact test). Adverse events are summarized elsewhere.^12^

## Discussion

This is the largest study to examine the effect of amantadine on cognitive performance in chronic TBI. In this chronic (>6 months) TBI sample with behavioral disturbance, both amantadine and placebo groups showed improvement on the cognitive measures and indices, with several memory measures demonstrating superior improvements in the placebo group at day 28. No significant group differences were found at day 60. Such significant placebo effects can be observed in chronic populations, but the difference suggests a marginally negative impact of amantadine on cognition.^22^

Both groups showed increases in normed scores on memory measures from baseline to day 28 and day 28 to day 60, likely attributed to practice effects. On most tests, the placebo group started from a nonsignificantly lower baseline. However, controlling for baseline, the placebo group showed slightly greater improvement on both GCI and LMI from baseline to day 28 compared to the treatment group, amounting to approximately 0.25 SD. However, by day 60, any difference in the amount of change from baseline between the two groups on memory measures was negligible. The most straightforward explanation could be that amantadine in the treatment group caused a temporary decrease in the expected practice effect on these memory measures, but by day 60, this temporary practice effect inhibition resolved so that both groups had equivalent practice effect from baseline to day 60. Alternatively, amantadine may have acted on limbic instability in this population with behavioral dysregulation, possibly downregulating behavioral and cognitive processing. In theory, amantadine might help mediate mesolimbic circuit regulation through dopaminergic mechanisms and impact behavior. However, the impact of amantadine on new learning and long-term potentiation is less clear given that these functions could be negatively impacted by amantadine's NMDA receptor antagonism.^23^ Other potential mechanisms include induction of depression and altered striatal plasticity.^24^ It is unlikely that a ceiling effect inhibited the practice effect in the amantadine group at 28 days given that this group continued to achieve higher scores at 60 days, and at 60 days reasserted their advantage observed at baseline.

The community-dwelling sample studied here was significantly impaired as demonstrated in Table 3. The mean on most tests for the sample is greater than 1.5 SD below the normative mean at baseline, suggesting that a substantial proportion of the sample scored even lower on these tests. It is possible that an extremely cognitively impaired population requiring residential care might respond to amantadine.

Although LMI improvement was superior for the placebo group, the effect may not have been specific to learning and memory. Learning and memory are dependent on other functions, such as distractibility or fatigue with extended cognitive tasks, which may have contributed to the transient reduction in cognitive functioning in the treatment group. Other than assuring that medications were not added or dosage changed during the study, we did not control for the use of other drugs, which may have affected our findings. Additionally, a constant challenge in chronic TBI intervention studies is the heterogeneous nature of the injuries. In future studies, it would be interesting to evaluate other markers of brain function, including blood flow or inflammation, to further characterize the participants and evaluate the brain's ability to respond to stimuli.

From a clinical standpoint, any neurostimulant or other neuromodulatory treatment may not be effective without additional directed therapy to practice the targeted cognitive ability. Amantadine has been shown to accelerate functional outcome acutely in minimally conscious state and vegetative state^3,4^ and improve irritable and aggressive behavior in chronic TBI.^12,25^ The risk-benefit profile of deciding on treatment will be different for each individual.

Taken together, these findings provide no evidence to support the prevailing belief that amantadine at 100 mg twice-daily dosing improves overall cognitive function, learning memory, attention, and processing speed in patients with chronic TBI, as measured by the neuropsychological measures used in this study. Only a couple of small studies have looked at the effect of amantadine on cognition previously. These earlier studies, using 200–400 mg/day, had mixed results. Along with our findings, studies to date provide no consistent evidence of amantadine's efficacy for improving overall cognitive function or specific aspects of cognitive function in individuals with complex mild-to-severe TBI. It would be useful, however, to reproduce this study in children and adolescents.

This study reports solely on changes on neuropsychological measures and does not include other means of function. The cohort's response of amantadine in respect to neurobehavioral function has been reported elsewhere.^12,25^ A study of the effect of amantadine on recovery of consciousness in patients with acute TBI (median 47 or 48 days post-injury)^3^ found that patients treated with amantadine showed more rapid improvement in responsiveness than those treated with a placebo, with no group differences after a washout period. A smaller study (*n* = 35) of individuals with acute TBI found greater improvements in Mini-Mental Status, Disability Rating Scale, GCS, and Functional Independence Measure who received amantadine 200 mg as compared to placebo for 6 weeks from time of injury to up to 12 weeks post-injury in a cross-over design.^26^ Perhaps there is a critical interval early post-injury during which treatment with amantadine improves consciousness even if this advantage is short-lived. Such an effect would not have been detected in the current investigation with randomization an average of 6 years post-injury. Certainly, arousal and consciousness differ from cognitive function, and amantadine would be expected to differentially impact these functions.

Of note, the date of injury for this cohort occurred up to as early as 6 months post-injury. Given that cognitive and neurobehavioral improvements may continue over the years post-injury, we do not know to what extent these individuals have or have not reached a cognitive and/or neurobehavioral plateau. Whereas the study enrolled individuals up to 6 months post-injury, the average time post-injury was 6.2 years (5.5 SD), with 46% more than 5 years post-injury.^27^ If the study had found positive changes post-injury, it is reasonable to ask how much is attributed to natural recovery versus how much is attributed to amantadine. However, in this study, which did not find positive change in cognitive function, the issue of ongoing recovery/change is not an explanation for our findings.

### Limitations

An evaluation of the effect of amantadine on cognition was not the primary focus of this study. Participants were not screened for a particular level of cognitive impairment. This resulted in a cohort that was probably performing at expectation for persons with history of complicated mild-to-severe TBI. If we had screened for persons with greater than typical cognitive impairment in the chronic period after TBI, our study may have provided a more useful test of the possible effect of amantadine on cognitive impairment. Additionally, by selecting those with behavioral disturbance, the benefit may have focused on behavioral dysfunction at the price of cognitive processing perhaps by regulating limbic function. Last, the heterogeneity of the group may have impacted the results, and those with either more-severe or less-severe patterns of deficits or specific biological patterns may have represented a better focused target.

## Conclusion

This is the largest known clinical trial to assess the effect of amantadine on cognitive function among individuals with chronic TBI. Amantadine 100 mg twice-daily in this population with chronic TBI appears not to be beneficial for overall cognition. Amantadine may have a small, transient negative impact on cognitive functioning during the first month of treatment. The use of amantadine to enhance cognitive function is not supported by the study findings.

## Supplementary Material

Supplemental data

## References

[1] Dikmen S.S., Corrigan J.D., Levin H.S., Machamer J., Stiers W., and Weisskopf M.G. (2009). Cognitive outcome following traumatic brain injury. J. Head Trauma Rehabil. 24, 430–438 19940676 10.1097/HTR.0b013e3181c133e9

[2] Warden D.L., Gordon B., McAllister T.W., Silver J.M., Barth J.T., Bruns J., Drake A., Gentry T., Jagoda A., Katz D.I., Kraus J., Labbate L.A., Ryan L.M., Sparling M.B., Walters B., Whyte J., Zapata A., and Zitnay G. (2006). Guidelines for the pharmacologic treatment of neurobehavioral sequelae of traumatic brain injury. J. Neurotrauma 23, 1468–1501 17020483 10.1089/neu.2006.23.1468

[3] Giacino J.T., Whyte J., Bagiella E., Kalmar K., Childs N., Khademi A., Eifert B., Long D., Katz D., Cho S., Yablon S.A., Luther M., Hammond F.M., Nordenbo A., Novak P., Mercer W., Maurer-Karattup P., and Sherer M. (2012). Placebo-controlled trial of amantadine for severe traumatic brain injury. N. Engl. J. Med. 366, 819–826 22375973 10.1056/NEJMoa1102609

[4] Whyte J., Katz D., Long D., DiPasquale M.C., Polansky M., Kalmar K., Giacino J., Childs N., Mercer W., Novak P., Maurer P., and Eifert B. (2005). Predictors of outcome in prolonged posttraumatic disorders of consciousness and assessment of medication effects: a multicenter study. Arch. Phys. Med. Rehabil. 86, 453–462 15759228 10.1016/j.apmr.2004.05.016

[5] Kraus M.F., Smith G.S., Butters M., Donnell A.J., Dixon E., Yilong C., and Marion D. (2005). Effects of the dopaminergic agent and NMDA receptor antagonist amantadine on cognitive function, cerebral glucose metabolism and D2 receptor availability in chronic traumatic brain injury: a study using positron emission tomography (PET). Brain Inj. 19, 471–479 16134735 10.1080/02699050400025059

[6] Reddy C.C., Collins M., Lovell M., and Kontos A.P. (2013). Efficacy of amantadine treatment on symptoms and neurocognitive performance among adolescents following sports-related concussion. J. Head Trauma Rehabil. 28, 260–265 22613947 10.1097/HTR.0b013e318257fbc6

[7] Schneider W., Drew-Cates J., Wong T., and Dombrovy M. (1999). Cognitive and behavioral efficacy of amantadine in acute traumatic brain injury: an initial double-blind placebo controlled study. Brain Inj. 13, 863–872 10579658 10.1080/026990599121061

[8] Leone H., and Polsonetti B.W. (2005). Amantadine for traumatic brain injury: does it improve cognition and reduce agitation? J. Clin. Pharm. Ther. 30, 101–104 15811161 10.1111/j.1365-2710.2005.00628.x

[9] Hammond F.M., Barrett R.S., Shea T., Seel R.T., McAllister T.W., Kaelin D., Ryser D.K., Corrigan J.D., Cullen N., and Horn S.D. (2015) Psychotropic medication use for traumatic brain injury patients during acute inpatient rehabilitation. Arch. Phys. Med. Rehabil. 96, 8 Suppl. 3, S2256–S2273 10.1016/j.apmr.2015.01.025 PMC4516906 26212402

[10] Albrecht J.S., Mullins D.C., Smith G.S., and Rao V. (2017). Psychotropic medication Use among medicare beneficiaries following traumatic brain injury. Am. J. Geriatr. Psychiatry 25, 415–424 28111062 10.1016/j.jagp.2016.11.018 PMC5365362

[11] Francisco G.E., Walker W.C., Zasler N.D., and Bouffard M.H. (2007). Pharmacological management of neurobehavioural sequelae of traumatic brain injury: a survey of current physiatric practice. Brain Inj. 21, 1007–1014 17891562 10.1080/02699050701559558

[12] Hammond F.M., Sherer M., Malec J.F., Zafonte R.D., Whitney M., Bell K., Dikmen S., Bogner J., Mysiw J., and Pershad R.; for the Amantadine Irritability Study Group. (2015). Amantadine effect on perceptions of irritability after traumatic brain injury. J. Neurotrauma 32, 1230–1238 25774566 10.1089/neu.2014.3803 PMC4523042

[13] Beck A.T., Steer R.A., and Brown G.K. (1996). Manual for the Beck Depression Inventory–II. The Psychological Corporation: San Antonio, TX

[14] Wechsler D. (1997). Wechsler Memory Scale-III. The Psychological Corporation: San Antonio, TX

[15] (1994). Army Individual Test Battery: Manual of Directions and Scoring. War Department, Adjutant General's Office: Washington, DC

[16] Benton A.L., and deS Hamsher K. (1989). Multilingual Aphasia Examination. AJA Associates: Iowa City, IA

[17] Delis D.C., Dramer J.H., Kaplan E., and Ober B.A. (2000). California Verbal Learning Test Second Edition (CVLT-II)—Adult Version Manual. The Psychological Corporation: San Antonio, TX

[18] Wechsler D. (1939). The Measurement of Adult Intelligence. Williams & Wilkins: Baltimore, MD

[19] Distribution of IQ Scores. MSN Encarta. http://encarta.msn.com/media_461540296/Distribution-of-IQ-Scores.html (no longer available) (last accessed 78, 2007)

[20] SAS. (2011). Statistical Analytical Software. SAS Institute Inc.: Cary, NC

[21] O'Brien P.C. (1984). Procedures for comparing samples with multiple endpoints. Biometrics 40, 1079–1087 6534410

[22] Polich G., Iaccarino M.A., Kaptchuk T.J., Morales-Quezada L., and Zafonte R. (2018) Placebo effects in traumatic brain injury. J. Neurotrauma. 4 5. doi: 10.1089/neu.2017.5506. [Epub ahead of print] PMC6016098 29343158

[23] Chow J.J., and Beckmann J.S. (2018). NMDA receptor blockade specifically impedes the acquisition of incentive salience attribution. Behav. Brain Res. 338, 40–46 29037660 10.1016/j.bbr.2017.10.013 PMC5681870

[24] Mancini M., Ghiglieri V., Bagetta V., Pendolino V., Vannelli A., Cacace F., Mineo D., Calabresi P., and Picconi B. (2016). Memantine alters striatal plasticity inducing a shift of synaptic responses toward long-term depression. Neuropharmacology 101, 341–350 26471421 10.1016/j.neuropharm.2015.10.015

[25] Hammond F.M., Malec J.F., Zafonte R.D., Sherer M., Bogner J., Dikmen S., Whitney M.P., Bell K.R., Perkins S.M., and Moser E.A. (2017). Potential impact of amantadine on aggression in chronic traumatic brain injury. J. Head Trauma Rehabil. 32, 308–331 28891908 10.1097/HTR.0000000000000342

[26] Meythaler J.M., Brunner R.C., Johnson A., and Novack T.A. (2002). Amantadine to improve neurorecovery in traumatic brain injury: a pilot double-blind randomized trial. J. Head Trauma Rehabil. 17, 300–313 12105999 10.1097/00001199-200208000-00004

[27] Hammond F.M., Hart T., Bushnik T., Corrigan J., and Sasser H. (2014). Changes and predictors of change in communication, cognition, and social function between 1 and 5 years after TBI. J. Head Trauma Rehabil. 19, 314–328 10.1097/00001199-200407000-00006 15263859

