# Supplementary material for: Amantadine Did Not Positively Impact Cognition in Chronic Traumatic Brain Injury: A Multi-Site, Randomized, Controlled Trial
Source: J Neurotrauma. 2018 Sep 24;35(19):2298–305. doi: 10.1089/neu.2018.5767 (PMC6157374; doi:10.1089/neu.2018.5767)
Supplement: Supplemental data [file Supp_Table.pdf]

Supplementary Data

Supplementary Digital Content Table. Sample-Size Inventory of Neuropsychological Measure Results Available for Each Group by Assessment Interval

	Baseline		*Day 28*		*Day 60*	
Measure	*Placebo*	*Amantadine*	*Placebo*	*Amantadine*	*Placebo*	*Amantadine*
Any neuropsychological data	60	59	58	55	57	52
Trials 1–5 Total Score (T-score)	60	59	58	55	57	52
Short Delay Free Recall (Z score)	60	59	58	54	55	52
Short Delay Cued Recall (Z score)	60	59	58	54	55	52
Long Delay Free Recall (Z score)	60	59	58	55	56	52
Long Delay Cued Recall (Z score)	60	59	58	55	56	52
Digit Span (Scaled score)	60	59	57	55	56	51
Processing Speed Index	60	59	58	55	56	52
Trail Making Test A (T-score)	60	59	58	55	56	52
Trail Making Test B (T-score)	60	59	57	55	56	52
COWAT (T-score)	60	59	58	55	56	52
General Cognitive Index	60	59	58	55	56	52
Learning/Memory Sub-Index	60	59	58	55	56	52
Attention/Processing Speed Sub-Index	60	59	58	55	56	52

COWAT, Controlled Oral Word Association Test.
